# TARGETING TRYPTOPHAN-KYNURENINE METABOLISM TO EXTEND LIFESPAN AND TREAT AGE-ASSOCIATED DISEASE

**DOI:** 10.1093/geroni/igz038.2294

**Published:** 2019-11-08

**Authors:** George L Sutphin, George L Sutphin

**Affiliations:** University of Arizona, Tucson, Arizona, United States

## Abstract

The kynurenine pathway, the major route for tryptophan catabolism, becomes dysregulated with age and in many age-associated pathologies in humans. Interventions targeting kynurenine metabolism are being pursued for neurodegeneration, cardiovascular disease, and chronic kidney disease. By manipulating kynurenine pathway enzymes and metabolites, we have extended lifespan up to 40% in Caenorhabditis elegans. Our most promising single target is the metabolite 3-hydroxyanthranilic acid dioxygenase (3HAA). Elevating physiological 3HAA by directly supplementing 3HAA or inhibiting the enzyme 3HAA dioxygenase (HAAO) extends worm lifespan by ~30% while reducing oxidative stress by directly degrading hydrogen peroxide. In rodents, anti-inflammatory activity of 3HAA improves outcomes in models of cardiovascular disease, asthma, and autoimmune encephalomyelitis. We are now beginning to validate our C. elegans work in mice and investigating a mechanistic model in which 3HAA acts to extend healthy lifespan by slowing age-associated accumulation of oxidative damage and repressing chronic inflammation.

